# Robotic assisted gait as a tool for rehabilitation of individuals with spinal cord injury: a systematic review

**DOI:** 10.1186/s12984-017-0338-7

**Published:** 2017-12-04

**Authors:** Ledycnarf J. Holanda, Patrícia M. M. Silva, Thiago C. Amorim, Matheus O. Lacerda, Camila R. Simão, Edgard Morya

**Affiliations:** 1 Neuroengineering Program, Edmond and Lily Safra International Neuroscience Institute, Santos Dumont Institute, Rodovia RN 160, Km 03, 3001 Distrito Jundiaí, Macaíba, 59280-000 Brazil; 2 Federal University of Rio Grande do Norte, Av. Sen. Salgado Filho Lagoa Nova, Natal, 59078-970 Brazil; 3 Anita Garibaldi Center of Education and Research in Health, Santos Dumont Institute, Rodovia RN 160, Km 02, 2010 Distrito Jundiaí, Macaíba, 59280-970 Brazil

**Keywords:** Robotic assisted gait, Rehabilitation, Robotic devices, Spinal cord injury

## Abstract

**Background:**

Spinal cord injury (SCI) is characterized by a total or partial deficit of sensory and motor pathways. Impairments of this injury compromise muscle recruitment and motor planning, thus reducing functional capacity. SCI patients commonly present psychological, intestinal, urinary, osteomioarticular, tegumentary, cardiorespiratory and neural alterations that aggravate in chronic phase. One of the neurorehabilitation goals is the restoration of these abilities by favoring improvement in the quality of life and functional independence. Current literature highlights several benefits of robotic gait therapies in SCI individuals.

**Objectives:**

The purpose of this study was to compare the robotic gait devices, and systematize the scientific evidences of these devices as a tool for rehabilitation of SCI individuals.

**Methods:**

A systematic review was carried out in which relevant articles were identified by searching the following databases: Cochrane Library, PubMed, PEDro and Capes Periodic. Two authors selected the articles which used a robotic device for rehabilitation of spinal cord injury.

**Results:**

Databases search found 2941 articles, 39 articles were included due to meet the inclusion criteria. The robotic devices presented distinct features, with increasing application in the last years. Studies have shown promising results regarding the reduction of pain perception and spasticity level; alteration of the proprioceptive capacity, sensitivity to temperature, vibration, pressure, reflex behavior, electrical activity at muscular and cortical level, classification of the injury level; increase in walking speed, step length and distance traveled; improvements in sitting posture, intestinal, cardiorespiratory, metabolic, tegmental and psychological functions.

**Conclusions:**

This systematic review shows a significant progress encompassing robotic devices as an innovative and effective therapy for the rehabilitation of individuals with SCI.

**Electronic supplementary material:**

The online version of this article (10.1186/s12984-017-0338-7) contains supplementary material, which is available to authorized users.

## Background

In the world, around 250 to 500 thousands people suffer spinal cord injury (SCI) every year. SCI is caused by a damage in neural structures of the spinal cord, such as medulla, medullary cone and/or cauda equina. Tissue lesions are mostly due to mechanical impact, laceration, compression, transection, infection or spinal cord degeneration. SCI is classified in two categories: incomplete or complete. The first is determined by motor and sensory partial preservation and reduced electromyographic (EMG) signal below the injured level, whereas the second is determined by a complete loss of sensorimotor activity and EMG signal below the level of lesion [1, 2]. The sensorial input and motor output are reduced or absent in SCI patients. Thus, muscular recruitment and motor planning become compromised, which leads to a reduced functionality [3, 4]. Chronic SCI patients are predisposed to secondary complications such as psychological, bowel, urinary, musculoskeletal, neuronal, cutaneous and cardiorespiratory [1, 4, 5]. Therefore, one of the challenges in neurorehabilitation to restore functional independence and quality of life is the recovery of planning and executing the movement again [2, 6]. Locomotor functional training is one of the rehabilitation aims in SCI patients. However, body weight sup- port (BWS) over a treadmill associated with manual assistance leads to therapist physical exhaustion. Since the exponential technological evolution, the idea of improving rehabilitation, and orthotic devices considering anatomic axes of lower limb have been developed. This may reduce professional physical exhaustion and bring new outcomes to rehabilitation [6, 7]. As a result, many studies that investigate the therapeutic effects of these orthotic devices have been published recently. Several researchers identified that robotic assisted gait training (RAGT) in SCI patients improved the cardiorespiratory, urinary, musculoskeletal, neuronal and somatosensory system, due to body compensation and neural plasticity [8–13]. Therefore, this review aims to compare features, and offer additional information for robotic devices application, and systematize scientific evidences from researches which used these devices as a tool for rehabilitation of SCI individuals. It is expected that this knowledge compilation may support further therapies, and researches to improve organic function and quality of life of SCI patients.

## Methods

### Literature search

The following databases were searched between February 2016 and October 2017: Cochrane Library, PubMed, PEDro and Periódico Capes and the manuscript was drafted from July 2016 to October 2017. To guarantee the most accurate keywords, it was conducted a search in Medical Subject Headings (MeSH) with “spinal cord injury”, “tetraplegic”, “paraplegic”, “lower limbs paralysis” and “lower extremity paralysis” associated with “body weight support”, “assisted gait”, “driven gait ortho- sis”, “orthotic gait training”, “gait training”, “robotic gait”, “exoskeleton”, “treadmill rehabilitation”, “walking”, “gait”, “locomotion”, “overground walking”, “robotic assisted gait”, “robotic assisted gait training”, “treadmill”, “physiotherapy”, “physical therapy”, “locomotion therapy”, “robotic assisted gait therapy”, “locomotor treatment”.

### Selection of trials

Two authors selected the relevant articles, according to the following pre-established criteria. Relevant researches were identified and included only publication in English, Portuguese and Spanish. Exclusion criteria: reviews, duplicates and not full article. All papers which used a robotic device for spinal cord injury rehabilitation were included independent of the publication year up to 2017.

## Data synthesis and results

**Fig. 1:**
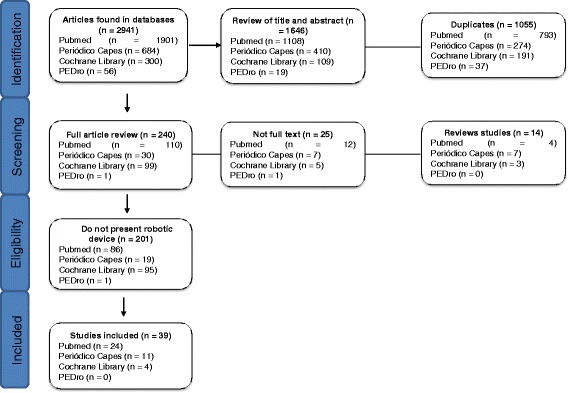
Prisma flowchart of the results from the literature search

The literature search undertaken yielded 2941 records, of which initially, the titles and abstracts were read and excluded the duplicate articles. In addition, review articles and not full text were excluded, resulting in 240 articles. Then, the articles that did not use robotic device were excluded. Thus, after careful analysis, 39 articles were included in the full-text review. Figure 1 illustrates the PRISMA flowchart of the results from the literature search performed by two authors followed guidelines for scale’s application as shown.

**Fig. 2:**
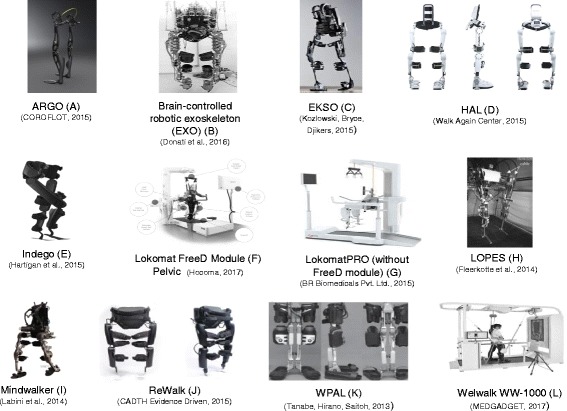
Robotic devices used for rehabilitation of individuals with SCI

**Fig. 3:**
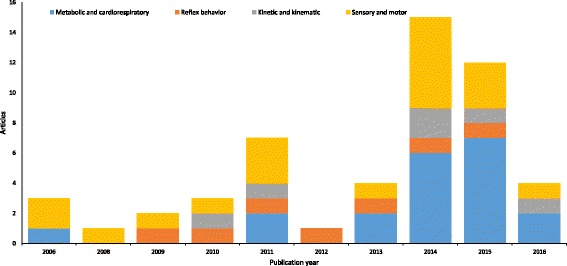
Robotic devices applications in the last years

Table 1 (see Additional file 1: Table 1) shows the robotic devices and their features e.g. treadmill, BWS, guidance force (GF), degrees of freedom (DOFs), maximum pa- tient weight and torso and upper limb muscle strength, and additional information available in the reviewed articles. All devices used in the reviewed articles are pre- sented in Figure 2 with exception of the Welwalk WW-1000 (K), which has not been published yet with SCI patient (see Figure: Figure 2). The Figure 3 (see Figure: Figure 3) schematics the increasing use of robotic devices over the years in rela- tion to the purpose of their application, highlighting the benefits of sensorimotor, cardiorespiratory, metabolic, reflex behavior; kinetic and kinematic parameters.

Further, the tables from two to five contain data from the articles: study design, patients’ demography, rehabilitation, outcome measures and results of each article inserted in this review listed according to the publication year in ascending order. Moreover, the tables were split into groups according to outcome measure: cardiorespiratory and metabolic parameters (Additional file 2: Table S2), parameters of reflex behavior (Additional file 3: Table S3), kinetic and kinematic parameters (Additional file 4: Table S4), and sensory and motors parameters (Additional file 5: Table S5) (see Additional file 2: Table S2, Additional file 3: Table S3, Additional file 4: Table S4, Additional file 5: Table S5, respectively).

## Discussion and conclusions

The primary goal of this study was to conduct a systematic review of scientific evidence related to the use and features of robotic devices in the rehabilitation of patients with SCI. From this review, it is possible to base and guide news prospects for rehabilitation and research, in view of the numerous benefits that this type of therapy is able to provide.

Robotic devices are technologies in constant evolution, e.g. recently was released the Welwalk WW-1000 (L), which allows pelvis, hip and knee motion for gait training with BWS and treadmill [14] (see Fig. 2). The nonstop innovation may offer a paradigm change in the treatment of individuals with a neurological disorder [15]. The exponential technological development lined up with rehabilitation device research is demanding new skills for the rehabilitation professional of the future.

The studies included in these review cited features in relation to the devices, which should be considered to select the appropriate tool for each individual due to the peculiarity of the requirements for its use. In addition, the devices cited may be ap- plied in indoor or outdoor environments, considering different DOFs, levels of GF, physical efforts demand and higher energy expenditure. The LokomatPRO (plus FreeD module) and LOPES are not portable and have as main specification indoor use, BWS and treadmill [16–19]. Whereas the ARGO, EKSO, Indego, Re- Walk and WPAL can be used either in outdoor and indoor environments. However, these equipment require high upper limb load, trunk muscles strength and excessive energy expenditure due to gait assistance devices such as crutches or walker to en- sure stability and safety of the user [9, 20–28]. Besides possessing these characteristics, the HAL and Mindwalker present a controlled activity by bioelectrical signals detected through surface EMG electrodes measured in extensor and flexor muscles of the hip and knee [12, 15, 18, 25, 26]. The EXO does not require neither muscles strength of upper limbs and trunk, nor postural control to execute gait and it is flexible to adjust in a variety of different leg lengths [11]. Thus, it seems reasonable that robotic gait technology will merge several features to allow innovative rehabilitation.

Moreover, this review showed that this type of intervention is able to provide benefits that exceed improvement of sensorimotor functions of this population. Figure 3 highlights the applications of robotic devices for improving sensorimotor, cardiorespiratory and metabolic parameters until the approaches to altered reflex behavior, kinetic and kinematic parameters (see Fig. 3). Among these changes are included, improvements in activity performance, postural control, urinary and bowel functions, which are directly related to the improvements of psychological characteristics and life quality.

The rehabilitation protocol based on specific task enhances neuroplasticity in individuals with SCI. In this case, it is important to consider the rehabilitation parameters used, which should be related to the injury level, classification, and the secondary changes. During any training session, the BWS level was selected as the minimum amount of assistance that would not create excessive knee flexion during posture or drag during the swing that was diminished by 5% of BWS. In the inserted studies, initial BWS was averaged 60% and lasted 45–50%. The weekly frequency predominance was 2–3 times per week with low to moderate intensity. The initial treadmill speed varied between 0.22 and 0.56 m/s and, increased by 0.02 m/s. Some studies have graded treadmill speed, GF and BWS according to HR, VO_2_ and MET of each individual.

Numerous studies revealed changes below the level of injury with multiple variables observed in the course of its protocol corroborating with results that indicate reduced pain perception and spasticity level [29, 30]. Likewise, it was observed changes in the proprioceptive ability, sensitivity to temperature, vibration, pressure, behavior reflex, electrical activity of muscle and cortical level [10, 11, 31–44] and changes in the level of injury classification. Further, recovery of sensorimotor function; increased stability and strength of muscles thoracic and lumbar region [20]; functional improvements in skills in sitting posture and gait, in the bowel and urinary function, cutaneous and psychological. Therefore, positively impacting the performance of their routine activities, making these individuals with higher level of functional independence [11].

One study [45] suggests that the RAGT associated with anabolic medicine does not promote alterations in bone mineral density (BMD) index, although another study [11] found divergent results revealing that half of the participants showed no change while the other half obtained a small evolution without relation to the improvement of neurological aspect.

Rehabilitation improvements of an individual with SCI involves cardiorespiratory fitness, which is important in improving the quality of life as well as contributing to a better performance in routine activities. It was observed that improvements in this aspect provide a better performance of gait related to increasing walking speed, stride length, distance walked [9, 24, 46–49], related to the reduction of HR, BF, MET and VO_2_. Studies [21, 31, 50, 51] found a VO_2_ and HR variation when compared these parameters in sitting and orthostatic positions. Further, it was observed that the higher GF applied to assist walking, the lower HR and MET, suggesting that the RAGT interferes in the increase of energy expenditure, this is capable to provide metabolic and cardiorespiratory benefits.

In conclusion, studies have been able to identify benefits promoted by the robotic gait devices in SCI treatments. It is expected in near future that devices should be used not only during therapy but also in daily living activities. None the less, the devices should be hybrid putting together the current SCI individuals abilities and supporting them where and when their skills lack, including feedbacks mechanisms to improve the user benefit from the device. It corroborates to a more individual centered therapy, based on their specifics needs. Scientific and technological evidence shows significant progress encompassing RAGT as an innovative and effective therapy for the rehabilitation of individuals with SCI, achieving promising results from improvements in cardiorespiratory, musculoskeletal, intestinal, urinary, neural, somatosensory and psychological functions. One of the important features of this type of treatment is to be a specific therapy based on motor learning improvements, in which promotes neuroplasticity and reduce secondary injury complications.

## Additional files

Additional file 1: Table S1. Description of the robotic device(s) used as a tool for rehabilitation of individuals with SCI. (PDF 157 kb)

Additional file 2: Table S2. Studies that presented metabolic and cardiorespiratory parameters as outcome measures in individuals with SCI. (PDF 257 kb)

Additional file 3: Table S3. Studies that presented altered reflex behavior parameters as outcome measures in individuals with SCI. (PDF 345 kb)

Additional file 4: Table S4. Studies that presented kinetic and kinematic parameters as outcome measures in individuals with SCI. (PDF 197 kb)

Additional file 5: Table S5. Studies that presented sensory and motors parameters as outcome measures in individuals with SCI. (PDF 273 kb)

## Abbreviations

10MWT: 10-meter walk test
2MWT: 2-minute walk test
6MWT: 6 min walk test
ABC: Activities-specific balance confidence
AH: Abductor hallucis
AIS: American Spinal Injury Association impairment scale
ARGO: Advanced reciprocating gait orthosis
AROM: Active range of motion
BDI: Beck depression inventory
BF: Breathing frequency
BFM: Biceps femoris
BMD: Bone mineral density
BWS: Body weight support
CF: Crutch force
CG: Control group
COPM: Canadian occupational performance measure
DELTa: Anterior deltoid
DELTp: Posterior deltoid
DF: Dorsi-flexors
DGO: Driven gait orthosis
DOFs: Degrees of freedom
DXA: Dual-energy x-ray absorptiometry
EAW: Exoskeletal - assisted walking
ECU: Extensor carpi ulnaris
EEG: Electroencephalogram
EG: Experimental group
EMG: Electromyographic
EXO: FAC: Functional ambulation category
FCU: Flexor carpi ulnaris
FES-1: Falls efficacy scale
FET: Figure eight scale
FIM: Functional independence measure
FSR: Force-sensitive resistors
GF: Guidance force
GMM: Growth mixture modeling
GRC: Gracilis
GTX: Graded exercise test
HAL: Hybrid assistive limb
HR: Heart rate
HS: Healthy subject
IPAQ: Impact on participation and autonomy questionnaire
LEMS: Lower extremity motor score
LH: Lateral hamstrings
LOA: Level of assistance
LSQ: Life satisfaction questionnaire
LTSO: Thoracolumbosacral orthosis
MCF: Mean crutch force
MeSH: Medical Subject Headings
MET: Metabolic equivalents
MG: Medial gastrocnemius
MH: Medial hamstrings
MVC: Maximal voluntary contraction
P1NP: Intact aminoterminal propeptide of type I procollagen
PCF: Peak crutch force
PCI: Physiological cost index
PF: Plantar-flexion
PL: Peroneus longus
ppSEP: Paired pulse somatosensory evoked potentials
PROM: Passive range of motion
PTH: Parathyroid hormone
RAGT: Robotic assisted gait training
RF: Rectus femoris
ROM: Range of motion
RPE: Rate of perceived exertion
SCI: Spinal cord injury
SCI-FAP: Spinal cord injury-functional ambulation profile
SCIM: Spinal cord independence measure
SHO: Sides of the acromium
SOL: Soleus
SpO_2_: Saturation of peripheral oxygen
SR: Spinal reflex
ST: Semitendinosus
TA: Tibialis anterior
TLSO: Thoracolumbosacral orthosis
TUGT: Timed up and go test
UEMS: Upper extremity motor score
VAS: Visual analog scale
VE: Pulmonary ventilation
VEL: Hip angular velocity
VL: Vastus lateralis
VM: Vastus medialis
VO_2_ peak: Peak cardiorespiratory capacity
VO_2_: Oxygen uptake
VP: Velocity peak
WISCI II: Walking index for spinal cord injury II
WPAL: Wearable power-assist locomotor
