# Supplementary material for: Robotic assisted gait as a tool for rehabilitation of individuals with spinal cord injury: a systematic review
Source: J Neuroeng Rehabil. 2017 Dec 4;14:126. doi: 10.1186/s12984-017-0338-7 (PMC5715997; doi:10.1186/s12984-017-0338-7)
Supplement: Supplementary file 1 — Description of the robotic device(s) used as a tool for rehabilitation of individuals with SCI. (PDF 157 kb) [file 12984_2017_338_MOESM1_ESM.pdf]

Table 1. Description of the robotic device(s) used as a tool for rehabilitation of individuals with SCI.

Robotic device (s)	Features						Additional information available in the reviewed articles
	Treadmill	BWS	GF	DOFs	Maximum patient weight	Requires torso and upper limb muscle strength	
ARGO (A) [9, 20].	O	O	●	●	—	●	❖ Excessive energy expenditure due to the use of devices to assist gait such as crutches to help walk.<br>❖ Gait pattern is not physiologic.<br>❖ Allows hip flexion of a lower limb to the lower contralateral member through a reciprocal bond.
Brain-controlled robotic exoskeleton (EXO) (B) [11].	O	O	●	12	—	O	❖ Can be associated with BWS, sensory feedback, and pressure sensors, wire sensors, gyroscopes and EEG.<br>❖ Designed to be anatomically coherent with the body of an individual, because hip-to-knee segments of the legs could be adjusted to accommodate a variety of different leg lengths.
EKSO (C) [22, 23, 26, 27].	O	O	●	4	—	●	❖ Passive spring-loaded ankle joints.<br>❖ Backpack that houses a computer.<br>❖ Battery supply.<br>❖ Wired controller.<br>❖ Provides support from the posterior pelvis to the upper back.<br>❖ A step will not be triggered unless crutches are firmly on the ground.<br>❖ Do not present severe orthostatic hypotension, significant cardiac or vascular disease and integumentary issues such as open wounds.<br>❖ No pregnancy.<br>❖ Do not present significantly decline of bone density as indicated by DXA or a history of pathological fractures.<br>❖ Patients which do not present bilateral upper-extremity strength, one functional upper extremity or one functional lower extremity.<br>❖ Patients with contractures greater than 10 in the hip or knee joint, leg length differences more than 2 cm or total hip replacements.

HAL (D) [12, 25, 26].	O	O	●	—	—	●	❖ Excessive energy expenditure due to a need for gait assistance devices such as crutches to help walk.<br>❖ Present a frame and robotic actuators that attach to the patient's legs.<br>❖ Joint movement is supported by electric motors.<br>❖ Initiate by minimal bioelectrical signals detected via surface EMG electrodes measured in hip and knee extensor and flexor muscles.<br>❖ Can be associated to treadmill and BWS.
Indego (E) [28, 31].	O	O	●	—	113 kg	●	❖ Enables to help sitting, walking, and standing as well as sit-to-stand, stand-to-walk, walk-to-stand, and stand-to-sit transitions or sit with 100% powered robotic assistance.<br>❖ Developed based on the user's ability to affect its center of pressure via the use of the upper body in combination with a stability aid.<br>❖ Excessive energy expenditure due to a need for gait assistance devices such as crutches to help walk.<br>❖ Height range 155 – 191 cm.<br>❖ Maximum hip width 42.2 cm.<br>❖ Femur length range 35 – 47 cm.<br>❖ Spasticity score: Modified Ashworth score 3 or lower.<br>❖ It is necessary sufficient upper body strength to balance and supports the forearms crutches, front-wheeled walker or platform walker.<br>❖ For complete and incomplete spinal cord injured individuals T4 or below.<br>❖ A Bluetooth LE radio allows communication between the Indego and iPhone or iPod touch through the custom Indego iOS application.
Lokomat FreeD Module (F) [19].	●	●	●	6	135 kg	O	❖ Patients can activate their core muscles and experience balance aspects.<br>❖ Video monitor up front.<br>❖ Allows lateral translation and transverse rotation of the pelvis.
LokomatPRO (without FreeD module) (G) [7, 10, 13, 29, 30, 32, 33, 34, 35, 36, 37, 38, 39, 40, 41, 42, 43, 44, 45, 46, 47, 48, 49, 50].	●	●	●	4	135 kg	O	❖ Gait pattern is physiologic.<br>❖ Compatible with pediatric orthoses.<br>❖ Video monitor up front.<br>❖ Used to measure isometric force (torque), the stiffness of the patient's joints while the legs are passively moved at 30, 60 and 90º/s, and PROM.

LOPES (H) [16, 17].	●	●	●	6	—	O	❖ Passive foot lifters can be added to keep dorsiflexion ankle.<br>❖ Video monitor up front.<br>❖ Severe contractures.<br>❖ Bone instability.<br>❖ Open skin lesions in the area of the lower limbs and torso.<br>❖ Cardiac and circulatory contraindications<br>❖ Severe cognitive deficits.<br>❖ Hip, knee, ankle arthrodesis.
Mindwalker (I) [15, 18].	O	O	●	6	100 kg	●	❖ Excessive energy expenditure due to a need for gait assistance devices such as crutches.<br>❖ Patient height between 1.53 - 1.88 m.<br>❖ Hip width up to 0.44 m.<br>❖ Requires high load on the upper limb joints.<br>❖ Can be associated to treadmill and BWS.<br>❖ Initiate by minimal bioelectrical signals detected via surface EMG electrodes measured in hip and knee extensor and flexor muscles.
ReWalk (J) [21, 23, 26].	O	O	●	—	100 kg	●	❖ Body height between 160 - 190 cm.<br>❖ Gait pattern is not physiological.<br>❖ Due to standing up/sitting down with the crutches, the device exerts pressure at the bend of the elbow and present risk of bruises.<br>❖ Requires walking aids (crutches or a walker) to ensure stability and safety of the user.<br>❖ The gait is a three-point pattern.<br>❖ Present a battery unit, computer contained in a backpack, wireless mode selector, sensors that measure upper-body tilt angle, joint angles, and ground contact.<br>❖ The exoskeleton is articulated to footplates distally and to a sacral band proximally.<br>❖ Additional modes include sit-to-stand, stand-to-sit, up and down stairs.

WPAL (K) [24].	O	O	●	6	136 kg	●	❖ The user will partially depend on crutches, or a walker to support their weight and maintain balance, joint angles and foot contact forces, which are often recorded to monitor basic gait performance.<br>❖ Exoskeleton and walker are placed at a convenient location suitable for walking (e.g. bedroom).<br>❖ Other robotic parts can be added by the user in the wheelchair.<br>❖ Robot can be folded into a roller bag and transported to anywhere.<br>❖ It can be used on uneven surfaces.

(•) Yes; (O) Not; (—) Not informed.
