# Supplementary material for: Robotic assisted gait as a tool for rehabilitation of individuals with spinal cord injury: a systematic review
Source: J Neuroeng Rehabil. 2017 Dec 4;14:126. doi: 10.1186/s12984-017-0338-7 (PMC5715997; doi:10.1186/s12984-017-0338-7)
Supplement: Supplementary file 2 — Studies that presented metabolic and cardiorespiratory parameters as outcome measures in individuals with SCI. (PDF 257 kb) [file 12984_2017_338_MOESM2_ESM.pdf]

Table 2. Studies that presented metabolic and cardiorespiratory parameters as outcome measures in individuals with SCI.

Study	Study design	Patients' demography	Rehabilitation	Outcome measures	Results
Kawashima et al., 2006 [9].	Cross-sectional, experimental research.	● 10 subjects with SCI.<br>● 1 female and 9 male.<br>● Aged between 25 and 34 years.<br>● Injury level: T5-T12.<br>● (AIS A and B<br>● Time lesion: 10 months and 3 years.	● ARGO.<br>● 10 weeks.	Cardiorespiratory evaluation: 3 min rest in standing position, followed by 20 min walk at a comfortable speed with telemetry following parameters: VE, VO_2 and HR.	In cardiorespiratory evaluation was observed that the higher the level of injury, the greater the values of the parameters evaluated when comparing the beginning and the end of the test.
Galen et al., 2011 [48].	Pilot study.	● 18 subjects with SCI.<br>● 4 female and 14 male.<br>● Aged between: 26 and 63 years.<br>● Injury level: C3-L1.<br>● AIS C and D.<br>● Time lesion between of 5 and 169 weeks.	● Lokomat.<br>● 8 weeks.<br>● BWS of 70-80%.<br>● Speed of 2.78-3.3 m/s, which was progressed according to the participant's tolerance.<br>● Based on participant's ability to maintain knee extension during mid-stance the BWS parameters were diminished, while the maximum speed increased was 5.56 m/s.	Evaluations were executed in the pre-intervention (first week), mid-intervention (fifth week) and post-intervention (last week).<br>● WISCI II.	In WISCI II scores, only 3 participants showed progression. In the evaluation of gait, 6 participants with acute SCI and 4 with chronic SCI were capable to walk 10 m without the assistance of orthosis or parallel bars. All participants showed progression in the initial foot contact, acute SCI participants presented the greater evolution resulting in statistically significant changes primarily in the period of pre and post-intervention.

Lam et al., 2011 [49].	Pilot study.	• 1 subjects with SCI.<br>• 1 male.<br>• Aged: 43 years.<br>• Injury level:T11.<br>• AIS D.<br>• Time lesion: 2 years.	• Lokomat.<br>• 45 minutes.<br>• 3 times per week.<br>• 12 weeks.<br>• At the beginning, the speed was 0.31 m/s and in the course of sessions, it was incremented by 0.03 m/s.<br>• Prior the increment, it was realized a warm-up period during 3-5 minutes with the treadmill speed based on the previous training session.<br>• The speed was incremented according to the patient's tolerance to a given speed, which was determined by its ability to maintain in the treadmill speed for a minimum of 15 min and by observing its ability to advance the feet beyond the hips.<br>It was monitored the training tolerance, in each training session.	• Borg CR10 scale.<br>• HR and BP changes.<br>• 10MWT.<br>• ABC.<br>• COPM.<br>Before and after the training program it was measured the tolerance to orthostatic stress.	According to training tolerance, the participants scored 4-7 in relation to limbs efforts and 3-5 referred to cardiorespiratory symptoms. The maximum HR exceeded 122 bpm, the saturation of peripheral oxygen (SpO_2) varied 94-98%, and the mean arterial pressure varied 75-113 mmHg. At the end of the rehabilitation protocol, it was observed a lower variation of HR and BP during the sit-up and stand-up. In the 10MWT, the patient's velocity increased from 0.18 m/s to 0.25 m/s, similarly in the 6MWT, the travelled distance increased from 58.8 m to 79.9 m, whereas it was observed no variations in the scores obtained in the WISCI II according to the initial evaluation.

Gordon, Wald and Schnitzer, 2013 [45].	Pilot study.	● 12 subjects with SCI.<br>● 2 female and 8 male.<br>● Mean aged: 34 years.<br>● Injury level: C3-L2.<br>● AIS A, B and C.<br>● Time lesion between 3 and 21 years.	● Only 9 of the 12 subjects received RAGT with Lokomat 3 days per week for 6 months.<br>● First session:<br>● 19 minutes.<br>● Speed: 4.44 m/s.<br>● BWS: 60%.<br>● Last session:<br>● 37 minutes.<br>● Speed: 5.83 m/s.<br>● BWS: 50%.<br>● During any given training session, the level of BWS was selected as the minimum amount of assistance that would not create excessive knee flexion during stance or toe drag during the swing.<br>● All the subjects received teriparatide 20 g/d, calcium 1000 mg/d, and vitamin D 1000 IU/d for the duration of the study.	● DXA of the spine, total hip, and femoral neck as well as micromagnetic resonance imaging.<br>● Laboratory tests.<br>The entire evaluation was performed with 3, 6, and 12 months RAGT.	During the 6 months of RAGT and the use of medicines, the changes observed for BMD, laboratory tests, and imaging showed low magnitude and little significant relevance. Therefore, further studies of longer duration and greater number of subjects is needed to assess the effects of anabolic steroids to improve the structural integrity of bone and bone strength.

Tanabe, Hirano and Saitoh, 2013 [24].	Pilot study, 1 group, pretest-posttest.	● 7 subjects with SCI.<br>● 1 female and 6 male.<br>● Mean aged: 46.29 years.<br>● Injury level: T6-T12.<br>● AIS A and B.<br>● Time lesion between 6 and 20 years.	● First stage: reinforcement exercise with parallel bars until reproducing robot movements. The step duration was 2s and the step length 0mm (stationary walking).<br>● Second stage: running exercise on the parallel bar. The step duration was 1.3 s and the step length 500 mm.<br>● Third stage: treadmill exercise with speed decreased to 0.09 m/s and the participant was instructed to perform lateral weight discharge over a longer continuous period.<br>● Last stage: walk exercise with a walker and without suspension. The patient commands the WPAL.	● Measured FAC, duration, and distance when walking with WPAL.<br>● HR monitoring.<br>● EMG activity from biceps brachii, triceps brachii, and trapezius during 6MWT.	Post-rehabilitation the participants were able to perform independent gait (FAC - 4). In addition, the walking time in the first evaluation varied between 5 and 8 minutes with bracing, and between 8 and 40 minutes with WPAL. The walking distance in the initial evaluation varied between 20 and 107 m (bracing) and between 30 and 640 m (WPAL). Further, the HR increased from 105 to 116 with WPAL and from 111 to 151 with the conventional orthosis. The ratio WPAL/conventional orthosis decreased: 44.7%, 87.8% and 70.9%, respectively. The results suggest that the reduction of the load in the upper limbs and the reduction in the increased HR may contribute to a longer gait over a longer distance.
Aach et al., 2014 [12].	Pilot study.	● 8 subjects with SCI.<br>● 2 female and 6 male.<br>● Aged between 36 e 63 years.<br>● Injury level: T8-L2.<br>● AIS A, B and C.<br>● Mean time lesion: 19 years.	● HAL.<br>● BWS of 50%.<br>● 5 times per week.<br>● 90 days.	● 10MWT.<br>● TUGT.<br>● 6MWT.	It was observed positive results in all evaluated parameters. It was observed a significant increase in velocity at 10MWT and 6MWT, and a decrease in TUGT from 55.34 s to 38.18 s.

Fenuta and Hicks, 2014 [50].	Pilot study.	● 7 subjects with SCI.<br>● Mean aged: 42 years.<br>● AIS C and D.<br>● Time lesion of 4 years.	● All the subjects used the same amount of BWS determined at the beginning session in the ZeroG.<br>● Subjects supported 3 walking sessions in BWS (between 30% and 41%).<br>● Andago GmbH treadmill system (with or without Lokomat) or overground ZeroG.<br>● Subjects concluded walking trials in a randomized order.	● Ergometric cycle to evaluate VO_2, HR, BF and rate of perceived exertion (RPE).<br>● Same parameters were used to observe the physiological response to the 2- minute training with Lokomat, and manual assistance and zero G training.	Comparing the Manual Treadmill or ZeroG session with the Lokomat sessions, the last one showed significantly lower HR, BF, VO_2, MET and muscle activity.
Fleerkotte et al., 2014 [16].	Pilot study.	● 12 subjects with SCI.<br>● 6 female and 6 male.<br>● Mean aged: 48.75 years.<br>● Injury level: C3-L2.<br>● AIS C and D.<br>● Time lesion between 14 and 122 months.	● LOPES.<br>● 8 weeks.<br>● 3 times per week.<br>● 60 minutes.<br>● Training intensity was incremented according to gait speed, training time and BWS.<br>● Gait speed enhanced from 0.43 to 0.58 m/s, the BWS (used only in five subjects) diminished from 8.5 to 7.4%.<br>● BWS diminished from 56.9 to 37.4%.	● 10MWT.<br>● 6MWT.<br>● TUGT.<br>● WISCI II.	Significant alterations were observed in 10MWT, 6MWT, although it was not observed in WISCI II, TUGT.

Hoekstra et al., 2014 [47].	Single-group pretest-posttest design.	● 10 subjects with SCI.<br>● 4 female and 6 male.<br>● Mean aged: 49 years.<br>● Injury level: C1-L2.<br>● AIS C and D.<br>● Time lesion less than 1 and 35 years.	● Each subject performed a GTX using an Angio arm ergometer.<br>● Prior the test, 5 minutes at rest, it was quantified the VO_2 and HR. The GTX consisted of 3 minutes of arm pedaling at 60 rpm in a minimum of three blocks.	● The therapist training:<br>● 24 sessions.<br>● 20-40 minutes.<br>● 10 to 16 weeks.<br>● The rehabilitation protocol consisted of a RAGT:<br>● Lokomat.<br>● 60 minutes.<br>● 2-3 times/week.<br>● The speed, device support and BWS were adapted to each individual, according to HR, VO_2 and MET.	The submaximal HR was significantly lower in the evaluation with cycle ergometer after training, suggesting a better cardiorespiratory fitness. In addition, most of the subjects performed minimum exercise intensity (<30% $VO_{2baseline}$, <30% $HR_{baseline}$ and <3 METs). The low-intensity exercise without changing the VO_2 on the ergometer cycle, the HR submaximal suggests some improvement in cardiorespiratory fitness. Therefore, training with robotic gait implies not only helps in gait rehabilitation but also secondary effects such as improvement in cardiorespiratory fitness. According to the first and the last tested training sessions, it was not found changes in VO_{2robot} and HR_{robot}.
Niu et al., 2014 [44].	Single-center, unblinded, and randomized study.	CG:<br>● 6 paraplegics and 14 tetraplegics.<br>● Mean aged: 49.7 years.<br>● Time lesion of 7.5 years.<br>EG:<br>● 40 subjects with SCI.<br>● 13 female and 27 male.<br>● Mean age: 42.2 years.<br>● Time lesion of 8.9 years.	● Lokomat.<br>● 4 days per week.<br>● 45 minutes.<br>● Speed of 0.94 m/s.	● 10MWT.<br>● TUGT.<br>● 6MWT.<br>● WISCI II.	It was used the Growth Mixture Modeling (GMM) to identify the effect of training on the groups and identify the group's growth trajectories. It was defined as low walking performance classes (Class 1) individuals that presented longer 10MWT and TUGT times, and shorter 6MWT distances at baseline. In the same way, it was defined as high walking capacity classes (Class 2) individuals that presented shorter 10MWT and TUGT times, and longer 6MWT distance. The 10MWT results shown beneficial changes when comparing pre- and post-treatment suggesting improvements in walking ability. The Lokomat group, Class 2 participants, increased walking speed by 0.033 m/s each week, while in the TUGT, only the participants of Class 2 of the Lokomat group reduced the time for performing the test (0.41 s per week), however, there were no significant changes in 6MWT in both groups.
Varoqui, Niu and Mirbagheri, 2014 [46].	Clinical trial.	● 30 subjects with SCI divided in two groups: 15 in the EG and 15 in CG.	● Lokomat.<br>● 3 times per week.<br>● 4 weeks.<br>● 30-45 minutes.<br>● BWS was diminished from 95% to 25%.<br>● Speed enhanced from 4.17 to 8.33 m/s.	● TUGT.<br>● 10MWT.<br>● 6MWT.	It was investigated the relation between the ambulation capability and the capability to realize voluntary movements with the ankle, thus it was found a significant correlation in 10MWT and TUGT It was not observed significant differences between, age, level and duration of lesion, WISCI II and PF muscle tone. The participants who performed TUGT and 10MWT in a shorter time and greater speed, showed improvement in gait speed and mobility, although it was not observed changes in 6MWT.

Arazpour et al., 2015 [20].	Quasi-experimental study.	● 4 subjects with SCI.<br>● 1 female and 3 male.<br>● Aged between: 20 and 32 years.<br>● Injury level: T8-T12.<br>● AIS A and B.<br>● Time lesion between 2 and 4 years.	● 5 days per week.<br>● 120 minutes.<br>● Between 6 and 8 weeks.<br>● The time of the gait training was divided into two parts, which the first part consisted of walk with the ARGO and the second with the ARGO/ TLSO.	● Polar HR monitor.<br>● Self-walking speed: the participants were required to walk along 40 meters, using an ARGO as a control condition or using ARGO/TLSO in a randomized order. It was calculated based on the walking distance and time according to 6MWT.	It was observed differences in the gait pattern in the comparison between ARGO and ARGO/TLSO. However, it was not statistically significant. In fact, the distance walked and gait velocity was shorter when walking with ARGO which may suggest that this is related to the position of the posture and trunk flexion of the participants in this type of orthosis, resulting in the increase of energy consumption to perform the task.
Asselin et al., 2015 [21].	Prospective, single-group observational study.	● 4 subjects with SCI.<br>● 1 female and 7 male.<br>● Mean aged: 37 years.<br>● Injury level: T1-T11.<br>● AIS A and B.<br>● Time lesion between 2 and 14 years.	● Rewalk without BWS.<br>● 40 sessions.<br>● 3 times per week.<br>● Speed between 0.22 and 0.27 m/s.<br>● 60-90 minutes.	● VO_2 and HR measured during sitting, standing and walking tests, consecutively for 6 minutes each.	During the walking, the VO_2 and HR were significantly higher when compared to sitting and standing positions. Furthermore, it was observed that the assistance level was inversely proportional to HR. The RAGT provided an increase in energy expenditure offering cardiorespiratory and metabolic benefits, and thus seems to be applicable to SCI individuals walk in daily lives.
Kozlowski, Bryce and Djikers, 2015 [22].	Longitudinal cohort design with a convenience sample.	● 7 subjects with SCI.<br>● Aged between 21 and 49 years.<br>● Injury level: C4-L1.<br>● AIS A, B and C.<br>● Time lesion between 0.4 and 7.4 years.	● EKSO.<br>● 20 minutes.<br>● Participant's progression less assistance was required, while the tolerance of longer walks time increased.<br>● It was incremented tasks that are more advanced for the participants perform, for example, going up and down ramps (up to 8% grade), walking on carpet and rough concrete surfaces.	● It was measured the HR and BP in sitting pre-session, right after upon sitting down post-session and in standing at mid-session.<br>● The RPE was measured in the standing position at all 3 points.	5 participants with minimal to moderate assistance walked up and down ramps and 5 in the same condition prior cited walked on a carpet with grades of 5 to 8%. The overall participants showed HR, systolic BP and RPE alterations during moderate intensity of exercises.

Evans et al., 2015 [31].	Pilot study.	● 5 subjects with SCI.<br>● 1 female and 4 male.<br>● Aged between: 28 and 51 years.<br>● Injury level: T6-T12.<br>● AIS A.<br>● Time lesion between 1 and 10 years.	● Not executed.	Two non-consecutive days were allocated for the evaluation.<br>● VO_2 peak: was used an arm cycle ergometer performing a GXT followed by two 6MWT using Indego with velocity between 0.19 and 0.27 m/s during 20 minutes.<br>● Evaluation cardiorespiratory and metabolic response at each walk test in the following conditions: 3 minutes of absolute rest in a sitting position, 3 minutes in a standing position, 6 minutes of assisted walking wearing an exoskeleton, repeating 3 minutes in a standing position. At the beginning and at the end of each 6MWT it was assessed the RPE by Borg's scale.	It was observed differences between walking velocity and total distance walked when compared the results of the first and the second 6MWT. The VO_2 peak and the metabolic response evaluated between the first 6MWT and standing position presented differences, nevertheless this was not considered significant.
Lam et al., 2015 [30].	Double-blind, stratified, randomized controlled trial design.	GC:<br>● 7 subjects with SCI.<br>● Aged between 26 and 55 years.<br>● Injury level: C2-T7.<br>● AIS C and D.<br>● Time lesion between 2 and 20 months.<br>EG:<br>● 8 subjects with SCI.<br>● Aged between 28 and 60 years.<br>● Injury level: C1-T10.<br>● AIS C and D.<br>● Time lesion between 2 and 20 months.	● 45 minutes.<br>● 3 times per week.<br>● 3 months.<br>● CG: Lokomat training without resistance.<br>● EG: Lokomat training with resistance.<br>● BWS established was the minimum tolerated by each participant with initial speed of 1 km/h and gradual increase 0.1 km/h according to the participant's capability of resist in the treadmill.	● Assessments were baseline, post-training, and follow-up (1 and 6 months).<br>● Borg scale.<br>● SCI-FAP.<br>● 10MWT.<br>● 6MWT.	In comparison to the baseline, the post-training SCI-FAP scores decreased by 204 points (95% confidence interval) in the Loko-R group and by 18 points in the CG. The 10MWT and 6MWT presented an increase across time, but there was no significant difference in these outcomes between groups. Further, compared to the baseline of all participants in both groups, the 10MWT increased by 0.01 m/s (95% CI: 0.05 to 0.16). The walking speed in the follow-up assessments, 1 month and 6 months, increased by 0.12 m/s (95% CI: 0.05 to 0.18) and 0.09 (95% CI: 0.03 to 0.15), respectively. Additionally, the 6MWT, post-training walking distance compared with baseline increased by 19.6 m (95% CI: 9.3 to 29.9), while at 1 month and 6 months follow-up assessments increased by 42.5 m (95% CI: 8.8 to 76.2) and 56.9 m (95% CI: 26.1 to 87.7), respectively.

Sczesny- Kaiser et al., 2015 [25].	Clinical trial.	GC:<br>● 11 HS.<br>● Mean aged: 27.8 years.<br>EG:<br>● 11 subjects with SCI.<br>● 4 female and 7 male.<br>● Mean aged: 46.9 years.<br>● Injury level: T7-L3.<br>● AIS A, B and C.<br>● Time lesion of 8.8 years.	● HAL.<br>● 30 minutes.<br>● 5 times per week.<br>● 12 weeks.	● It was performed 10MWT pre and post each treatment session, detecting time, numbers of steps and necessary assistance to complete the test.<br>● TUGT.<br>● 6MWT.<br>● LEMS.	There were also significant differences between the results of initial and post-treatment functional tests (TUGT, 6MWT and 10WTM and LEMS) of the SCI and HS group.
Yang et al., 2015 [51].	Pilot study.	● 12 subjects with SCI.<br>● 2 female and 10 male.<br>● Mean aged: 46 years.<br>● Injury level: C8-T10.<br>● AIS A, B and C.<br>● Time lesion of 6.8 years.	● Rewalk.<br>● RAGT time progressed from 60 to 120 minutes.<br>● 55 sessions.<br>● Prior gait training it was necessary that all participants established proficiency in standing balance, weight shifting, and sitting and standing transfers. In the standing position, it was required to participants show independent weight shifting with corresponding crutch placement in a 360° circumference.<br>● It was used a full-length freestanding mirror to provide the visual feedback and assist in the postural adjustments.	● 10MWT.<br>● 6MWT.<br>● LOA.<br>● Analysis of posture and gait pattern.	The overall results to HR and BP were within the expected ranges for rest, standing and EAW. All participants were capable to ambulate at a speed of 0.40 m/s; this speed leads for outdoor activity related community ambulation. Further, 5 participants conducted the 6MWT and 10MWT in an independent manner, 3 participants conducted these tests with nearby assistance, 3 conducted with minimal assistance and 1 conducted with moderate assistance. Finally, it was observed significant inverse relationships between EAW and LOA velocity for both tests (6MWT and 10MWT).

Donati et al., 2016 [11].	Pilot study	● 8 subjects with SCI.<br>● 2 female and 6 male.<br>● Aged between: 26 and 38 years.<br>● Injury level: T4-T11.<br>● AIS A and B.<br>● Time lesion between of 3 and 13 years.	At the end, the total of sessions was 2,052 equivalent to 1,958 hours. The protocol consisted of conventional physiotherapy and BMI application paradigms contemplated 6 items:<br>● Patient seated inserted in a virtual environment, using its own brain activity (captured by 16 EEG channels).<br>● Immersion in a virtual environment and similar BMI protocol while the patient was standing by a stand-in- table device.<br>● Training on Lokomat.<br>● Training with a BWS gait system fixed on an overground track by ZeroG.<br>● Training with a EXO, BWS gait system on a treadmill.<br>● Gait training with a brain-controlled, sensorized 12 DOFs robotic exoskeleton.<br>Only in items 3 and 4 individuals did not receive tactile-visual feedback.	On the first day of patients, training started the clinical assessments (Day 0), which was repeated after, 4, 7, 10 and 12 months.<br>● WISCI II.<br>● SCIM.<br>● BDI.<br>● BMD	One way of demonstrating how a motor function recovery has a positive effect on WISCI II functionality in the 5 months represents significant improvements in walking skills; two patients increased their performance in six levels, four patients in five levels and two patients in three levels. Moreover, it was noticed improvements in gastrointestinal function and overall appearance of the skin. In addition, 50% of the patients maintained a stability of the BMD, while the other 50% showed a slight evolution unrelated to the neurological recovery. In the SCIM questionnaire was observed, which all patients had a good level of independence in daily activities at the beginning of treatment, and after 12 months, some patients were able to improve.

Sale et al., 2016 [27].	Pilot single case experimental (pre and posttest) design study.	● 3 subjects with SCI.<br>● 1 female and 2 male.<br>● Aged between: 21 and 50 years.<br>● Injury level: T10-L1.<br>● AIS A and C.	● EKSO.<br>● 50 minutes.<br>● 2-4 days per week.<br>● 20 sessions.<br>Full training was divided into 4 modalities:<br>● Physical therapist actuates steps with a button push, called FirstStep mode;<br>● Mode the user takes control of actuating their steps via buttons on the crutches or walker, called ActiveStep mode;<br>Participants move hips forward and shifting them laterally, and the steps are triggered by the user's weight shift plus the initiation of forwarding leg movement (Pro Step).	● 6MWT.<br>● Participant Satisfaction Questionnaire.<br>● VAS.<br>● Indoor and outdoor TUGT.<br>● Borg Scale.	During the protocol, the participants presented a greater tolerance to the effort, since the assigned score decreases 36%, reduction of the Fatigue and Pain VAS scores corresponding to 27% and 9%, respectively. Regarding the functional tests, it was observed that the TUGT time decreased 44%, suggesting an improvement in the functional mobility of the participants, the indoor performance of 6MWT showed an improvement of 41% and outdoor of 102.78%.
