# Supplementary material for: Robotic assisted gait as a tool for rehabilitation of individuals with spinal cord injury: a systematic review
Source: J Neuroeng Rehabil. 2017 Dec 4;14:126. doi: 10.1186/s12984-017-0338-7 (PMC5715997; doi:10.1186/s12984-017-0338-7)
Supplement: Supplementary file 3 — Studies that presented altered reflex behavior parameters as outcome measures in individuals with SCI. (PDF 345 kb) [file 12984_2017_338_MOESM3_ESM.pdf]

Table 3. Studies that presented altered reflex behavior as outcome measures in individuals with SCI.

Study	Study design	Patient's demography	Rehabilitation	Outcomes measures	Results
Dietz et al., 2009 [32].	Case series.	CG:<br>● 10 HS.<br>● Mean aged: 38.2 years.<br>EG:<br>● 39 subjects with SCI.<br>● 3 female and 36 male.<br>● Mean age: 37.81 years.<br>● Injury level: C3-L3.<br>● AIS A, B and C.<br>● Time lesion: 3 months and 14 years.	● Lokomat.<br>● BWS of 65-75%.<br>● Constant speed at 0.56 m/s.<br>● 10-15 minutes.<br>● SR: evoked with the electrical stimulator AS 100 current source which presents similar stimulus intensity at the right distal tibial nerve at the dorsal aspect of the medial malleolus. This electrical stimulus presents a train of eight biphasic rectangular pulses with frequency of 200Hz and duration of 2 ms with a total period of stimulation of 40 ms. The intensity was set based on the first contraction of the AH muscle.	It was analysed leg muscle activity during walking with DGO, using EMG surface electrodes from RF, MG, TA and BFM.	The data was shown according to the EMG activity during 2 and 10 minutes of training in 3, 6 and 41 months after SCI injury. The EMG signal progressively enhanced until 3-4 months after spinal shock period, while there was a decrease in BFM, RF and MG. Approximately 6 months after SCI injury, the participants showed a degree of decline in the EMG amplitude and 41 months later showed a marked reduction. It was observed, a great diminish in signal amplitude in MG and BFM. In the SCI acute phase, it was observed a component of short latency 85 ms, and with 6 and 41 months 220 ms in TA, which is noticed a significant difference when the signal is compared prior and after the lesion. After 1-1.5 years post SCI injury, the TA activity is reduced to almost zero in the majority of SCI patients training.

Bolliger et al., 2010 [10].	Clinical trial.	CG:<br>● 10 HS.<br>● Mean aged: 38.2 years.<br>EG:<br>● 39 subjects with SCI.<br>● 3 female and 36 male.<br>● Mean age: 37.81 years.<br>● Injury level: C3-L3.<br>● AIS A, B and C.<br>● Time lesion: 3 months and 14 years.	● Lokomat in a constant speed at 0.56 m/s.<br>● Electrical stimulator was similar to [32].	The EMG activity was recorded from RF, GM, BFM and TA. The measurements are comprised of 70 and 100% BWS in 3 positions organized randomly in 6 conditions. In the TA muscle of the ipsilateral stimulated leg, it was analysed the SR activity as the reflex response which is reliable when observed in the TA muscle.	In all conditions of the CG, consistent early SR components appeared in the TA muscle (mean latency of 75 ms). In different conditions, there are not differences in the early SR components. However, the late SR components presented more consistently and significant considerable during the locomotion in comparison to the static condition (mean latency of 173 ms). Therefore, the late reflex component was significantly greater in the dynamic condition when compared to the static, whereas the early SR component remained unchanged overall conditions. In the static upright position, the SCI participants presented primary late SR components (mean latency of 214 ms). Among different conditions, there are not differences in the BWS compared to background activities.
Hubli, Dietz and Bolliger, 2011 [34]	Clinical trial.	CG:<br>● 10 HS.<br>● Mean aged: 40 years.<br>EG:<br>● 20 subjects with SCI.<br>● 3 female and 17 male.<br>● Aged between: 23.6 and 56.3 years.<br>● Injury level: C4-T11.<br>● AIS A and B.<br>● Time lesion: 3 months and 17 years.	● Lokomat.<br>● BWS of 70%.<br>● Speed constant at 0.56 m/s.<br>● Electrical stimulus presented a train of eight biphasic rectangular pulses with frequency of 200 Hz and duration of 2 ms. In a sitting position was determined the SR threshold according to a gradual increase in stimulation intensity with 2 mA steps. Stimulation intensity of HS was increased until it was observed the first movement of the hallux, when reached this; the	During assisted locomotion in Lokomat, EMG activity was recorded from bilateral proximal and distal leg muscles (RF, MG, BFM and TA).	The SCI participants were divided into 2 groups conforming to SR behavior along the training. The 2 groups consisted of 15 SCI participants which presented dominant early SR behavior (mean of 0.64) and 5 SCI participants which presented dominant late SR behavior (mean of 0.87). Moreover, the mean SR behavior of the HS was 0.77 and there are not significant differences between the early and the late group in terms of duration of lesion, level of lesion and age. The

			intensity was doubled for SR during assisted walking (8 – 18 mA). This was necessary for stabilization of SR elicitation during assisted locomotion of SCI participants. Thus, the intensity of stimulation was determined 2 times the reflex threshold (TA muscle response in EMG of 10 – 40 mA).		SR responses shortly interrupted the locomotor EMG pattern, resulting in a short BFM and TA activation and a slight inhibition of GM activity, subsequently, this interruption the normal locomotor EMG pattern continues. Similarly, it was observed in the 15 SCI participants with dominant early SR behavior, whereas in the 5 participants with dominant late SR behavior, the locomotor EMG pattern presented a long-lasting RF and TA muscles activation.
Mirbagheri et al., 2012 [35].	Pilot study.	● 12 SCI subjects.<br>● Presented incomplete motor function loss.	● Lokomat.<br>● 3 days per week.<br>● 45 minutes.<br>● 4 weeks of training.<br>● Speed was set approximately 2.78 m/s or lower and therapist increased it up to 6.94 m/s according to participant tolerance.	● Assessment of torque using a custom joint stretching apparatus when the participant's ankle was exposed to position perturbation during flexion or extension ankle position.<br>● Potentiometer, tachometer and torque transducer allowed the measurement of joint position, velocity and torque, respectively.<br>● Bipolar surface electrodes of EMG were recorded the TA and MG activity.	Passed 4 weeks of training, the reflex and intrinsic stiffness were significantly reduced, showing that the treatment proposed allowed benefits for neuromuscular properties. Under these circumstances, it was suggested the effectiveness of the Lokomat in the rehabilitation of SCI patients.

Knikou, 2013 [36].	Pilot study.	● 14 subjects with SCI.<br>● 4 female and 10 male.<br>● Mean aged: 39 years.<br>● Injury level: C5-T10.<br>● AIS A, B, C and D.<br>● Time lesion between 0.5 and 8 years.	● Lokomat.<br>● 45 sessions.<br>● 5 days per week.<br>● AIS A-B participants, first session:<br>● BWS: 60%.<br>● Speed: 0.44 m/s.<br>● AIS C-D participants, first session:<br>● BWS: 40%.<br>● Speed: 0.55 m/s.<br>● In the subsequent sessions, the BWS was diminished by 5% and the speed increased by 0.02 m/s.<br>● If quadriceps and triceps surae strength increased by a full score, the BWS was diminished 10% in each session. The ankle braces straps were loosened or tighten based on the TA muscle strength, which was assessed every 3-5 training sessions.	● Bilateral EMG activity from the following muscles: VL, GRC, LH, MH, TA, PL, MG and SOL.	This rehabilitation provided a functional reorganization of the H-reflex of the SOL. After training, at heel contact, late stance, mid and late swing phase and at swing-to-stance transition phase, it was observed the decrease of the SOL H-reflex from the right leg in AIS A-B participants compared to the H-reflex prior training. In the same way, after training, at late stance, swing phase initiation and mid- swing phases also it was observed a decrease of the right SOL H-reflex in AIS C-D participants compared to prior training. Furthermore, it was detected that after training, the BWS required for stepping without knee collapse diminished significantly, with an overall percentage change of 50, 41 and 74.2% for AIS A–B, AIS C, and AIS D subjects, respectively.

Smith et al., 2014 [37].	Clinical trial.	CG:<br>● 10 HS.<br>● Mean aged: 38.2 years.<br>EG:<br>● 9 subjects with SCI.<br>● 2 female and 7 male.<br>● Mean Age: 36.8 years.<br>● Injury level: C5-T10.<br>● AIS A, C and D.<br>● Time lesion: 0.5 and 11 years.	● Lokomat.<br>● 60 minutes.<br>● 5 days per week.<br>● AIS A participant:<br>●Started the training with BWS at 60% and at 4.39 m/s of speed.<br>●In the next subsequent sessions, the BWS was diminished by 5% and the speed increased by 0.19 m/s.<br>● AIS C and D participant:<br>●Started the training with BWS at 40% and at 5.5 m/s of speed.<br>●In the next subsequent sessions, the BWS was diminished by 10% and the speed increased by 8.28 m/s.<br>● It was determined that the lowest stimulation intensity that evoked EMG response in the ipsilateral TA without joint or limb movement is the reflex threshold during BWS in standing position. This intensity encompasses from 37 to 110 mA in HS participants, and it was 3.15 times the sensory threshold. The SCI participants, prior locomotor training, the sural nerve stimulation was 1.1 to 1.5 times the reflex threshold and after locomotor training was 1.05 to 1.46 times the reflex threshold.	SR assessments: it was observed and analysed changes in the flexor reflex behavior during gait of chronic SCI individuals.	SCI individuals presented modifications in the excitability of medullary pathways, which mediate primary flexor reflexes and increase the excitability of these pathways. The phase dependent modulation phase of the H-reflexes is re-established, the homosynaptic facilitation is reversed for homosynaptic depression, which inhibition is exerted at the motoneuronal level and improved in locomotor training. In addition, promote spinal integration. The RAGT contributes to the appearance of the first TA flexor reflexes during gait in accordance with the reduction of the amplitude in both lower limbs. This promotes a dependent modulation of these reflexes phase.

Mirbagheri, Kindig and Niu, 2015 [38].	Clinical trial.	CG:<br>● 23 subjects with SCI.<br>● Mean aged: 47.9 years.<br>● Time lesion: 8.9 years.<br>EG:<br>● 23 subjects with SCI.<br>● 7 female and 16 male.<br>● Mean Age: 36.8 years.<br>● Time lesion: 10.1 years.	The RAGT group rehabilitation:<br>● 3 times per week.<br>● 60 minutes.<br>● 4 weeks.<br>● The training progression was according to individual's ability, which consisted of increase treadmill speed from 1.5 to 2.8 km/h, diminish the robotic assistance of each leg to 20% of assistance, and diminish the BWS from 95% to 25%.	● The ankle joint neuromuscular properties were quantified by ankle perturbations to elicit the stretch reflex. The individuals were seated in an experimental chair where the tested ankle was strapped and mounted in a custom foot rest to the shaft of a position-controlled servo motor, aligned with the ankle joint center of rotation. A rotary encoder and 6-axis torque transducer recorded ankle position and torque, respectively. It was determined the neutral position as 90° and plantarflexion negative.<br>● EMG activity was measured on TA and MG.<br>The PROM was measured before the experiment that consisted of an examiner manually stretched the individual's ankle until reach pain or maximal resistance. Furthermore, it was applied a pseudorandom binary sequence perturbation to the ankle by the motor, with amplitude of 0.03 rad, switching rate of 150 ms, at positions increments of 5° over its PROM. This was performed with the knee held at 60° flexion.<br>● The evaluations were repeated for comparisons prior and after (1, 2 and 4 weeks of RAGT).	It was observed that the RAGT reduced peak magnitude and stiffness offset, in the same way, reduced its modulation related to joint angle for both. Overall, the intrinsic and reflex parameters increased, while spasticity and plantar flexor reflex excitability decreased. Thus, it was suggested that long-term training appear to benefit SCI individuals.
