# Supplementary material for: Robotic assisted gait as a tool for rehabilitation of individuals with spinal cord injury: a systematic review
Source: J Neuroeng Rehabil. 2017 Dec 4;14:126. doi: 10.1186/s12984-017-0338-7 (PMC5715997; doi:10.1186/s12984-017-0338-7)
Supplement: Supplementary file 4 — Studies that presented kinetic and kinematic parameters as outcome measures in individuals with SCI. (PDF 197 kb) [file 12984_2017_338_MOESM4_ESM.pdf]

Table 4. Studies that presented kinetic and kinematic parameters as outcome measures in individuals with SCI.

Study	Study design	Patients' demography	Rehabilitation	Outcome measures	Results
Gordon et al., 2010 [33].	Pilot study	● 10 subjects with SCI.<br>● 2 female and 8 male.<br>● Aged between: 26 and 63 years.<br>● Injury level: C3-L1.<br>● AIS C and D.<br>● Time lesion between 5 and 169 weeks.	● Lokomat.<br>● BWS of 100%.<br>● The participant was lifted 25 cm above treadmill surface at 0.55 m/s speed.	During stepping, it was evaluated EMG activity, lower limb joint kinematics and kinetics. In addition, using the Lokomat joint sensors it was assessed hip and knee joint kinematics. The EMG signals from the SOL muscles were recorded.	The results of this study suggested a significant increase in the signal amplitude of the SOL in the EMG and the load application. This implied that the capacity of locomotors and nervous adaptations to different efferent patterns through the combination of feedforward and feedback strategies. Further, the participants used different patterns to control the hip and ankle joints to provide these modulations.
Galen et al., 2011 [48].	Pilot study.	● 18 subjects with SCI.<br>● 4 female and 14 male.<br>● Aged between: 26 and 63 years.<br>● Injury level: C3-L1.<br>● AIS C and D.<br>● Time lesion between 5 and 169 weeks.	● Lokomat.<br>● 8 weeks.<br>● BWS of 70-80%.<br>● Speed of 2.78-3.3 m/s.<br>● Based on participant's ability to maintain knee extension during mid-stance the BWS was diminished, while the speed increased to 5.56 m/s.	● LEMS of the following muscles: hip flexors, knee extensors, ankle dorsiflexors, ankle plantar flexors and long toe extensors.	At the beginning of the study, 3 participants were scored with AIS C and the end progressed to AIS D. In this same way, at the end all participants diminished the BWS level. According to LEMS results obtained, acute SCI participants presented greater evolution in comparison to chronic participants.
Fleerkotte et al., 2014 [16].	Pilot study.	● 12 subjects with SCI.<br>● 6 female and 6 male.<br>● Mean aged: 48.75 years.<br>● Injury level: C3-L2.<br>● AIS C and D.<br>● Time lesion between 14 and 122 months.	● LOPES.<br>● 8 weeks.<br>● 3 times per week.<br>● 60 minutes.<br>● Training intensity was incremented according to gait speed, training time and BWS.<br>● Gait speed enhanced from 0.43 to 0.58 m/s, the BWS (used only in five subjects) diminished from 8.5 to 7.4%.<br>● BWS diminished from 56.9 to 37.4%.	● LEMS.<br>● Spatiotemporal parameters.<br>● ROM of the knee and hip.	It was observed significant modifications in the majority of spatiotemporal parameters. In contrast, there were not significant changes in the maximum knee flexion in the swing phase, the knee ROM in the stance phase, and the step width. The mean changes in the weak leg exceeded the changes in the strong leg, this was observed in the hip ROM and step length. Finally, significant alterations it not observed in LEMS.

Varoqui, Niu and Mirbagheri, 2014 [46].	Clinical trial.	● 30 subjects with SCI divided in two groups: 15 in the EG and 15 in CG.	● Lokomat.<br>● 3 times per week.<br>● 4 weeks.<br>● 30-45 minutes.<br>● BWS was diminished from 95% to 25%.<br>● Speed enhanced from 4.17 to 8.33 m/s.	● TUGT.<br>● 10MWT.<br>● 6MWT.	The 6MWT applied in order to assess walking endurance capacity showed significant correlation to AROM, VP, $AROM_{1stMU}$ and MVCDF. It was not observed significant differences between, age, level and duration of lesion, WISCI II and PF muscle tone. In contrast, the Lokomat group presented a significant improvement in relation to ankle AROM, however, this improvement was not observed in the CG. The kinematics parameters AROM, VP and $AROM_{1stMU}$ were used in the quantitative evaluation of DF voluntary movement, the results consisted of a statistically significant improvement in the Lokomat group, which means that this group was capable to move the ankle voluntarily at a greater distance. These represent an improvement of the kinematics components evaluated, which can be related to significant evolution in the muscle strength of PF and DF, according to MVC.
Kozlowski, Bryce and Djikers, 2015 [22].	Longitudinal cohort design with a convenience sample.	● 7 subjects with SCI.<br>● Aged between 21 and 49 years.<br>● Injury level: C4-L1.<br>● AIS A, B and C.<br>● Time lesion between 0.4 and 7.4 years.	● EKSO.<br>● 20 minutes.<br>● Participant's progression less assistance was required, while the tolerance of longer walks time increased.<br>● It was incremented tasks that are more advanced for the participants perform, for example, going up and down ramps (up to 8% grade), walking on carpet and rough concrete surfaces.	● It was evaluated the number of sessions required to achieve a rating of minimal assistance, support only for standing/sitting and for walking; to measure walking tolerance and physical effort. The walking tolerance was evaluated according to walk time, time spent taking steps and standing, a number of steps and approximate distance walked during participant's longest walk and the distance walked in the 2MWT.	Among the participants, only 6 were capable to walk at minimal assistance during 8 sessions and were capable to stand/sit with minimal assistance. While in approximately 15 sessions, 5 of these participants achieved support when necessary or close supervision assistance and were capable to stand/sit with contact guard assistance. The number of steps during the long training varied between 561 and 2616 executed between 28 and 94 minutes, with 57-107 minutes of standing training. The distance performed in the 2MWT was between 13.8-24.9 m with a mean speed of 0.11-0.21 m/s.

Sale et al., 2016 [27].	Pilot single case experimental (pre and posttest) design study.	● 3 subjects with SCI.<br>● 1 female and 2 male.<br>● Aged between: 21 and 50 years.<br>● Injury level: T10-L1.<br>● AIS A and C.	● EKSO.<br>● 50 minutes.<br>● 2-4 days per week.<br>● 20 sessions.<br>Full training was divided into 4 modalities:<br>● Physical therapist actuates steps with a button push, called FirstStep mode;<br>● Mode the user takes control of actuating their steps via buttons on the crutches or walker, called ActiveStep mode;<br>Participants move hips forward and shifting them laterally, and the steps are triggered by the user's weight shift plus the initiation of forwarding leg movement (Pro Step).	● Spatiotemporal parameters were evaluated at 3D gait.	Through the initial and final evaluation of the space-time parameters, an increase in gait velocity was observed, which occurred due to improvements in cadence and step length. During the protocol, the participants presented a greater tolerance to the effort.
