# Supplementary material for: Robotic assisted gait as a tool for rehabilitation of individuals with spinal cord injury: a systematic review
Source: J Neuroeng Rehabil. 2017 Dec 4;14:126. doi: 10.1186/s12984-017-0338-7 (PMC5715997; doi:10.1186/s12984-017-0338-7)
Supplement: Supplementary file 5 — Studies that presented sensory and motors parameters as outcome measures in individuals with SCI. (PDF 273 kb) [file 12984_2017_338_MOESM5_ESM.pdf]

Table 5. Studies that presented sensory and motors parameters as outcome measures in individuals with SCI.

Study	Study design	Patients' demography	Rehabilitation	Outcome measures	Results
Kawashima et al., 2006 [9].	Cross-sectional, experimental research.	● 10 subjects with SCI.<br>● 1 female and 9 male.<br>● Aged between 25 and 34 years.<br>● Injury level: T5-T12.<br>● AIS A and B.<br>● Time lesion: 10 months and 3 years.	● ARGO.<br>● 10 weeks of training.	Gait assessment: walk in a 10 m hall and analysis of the three- dimensional motion with VICON system.	It was observed a strong relationship between the cardiorespiratory evaluation parameters and the performance of the gait. Moreover, 80% of individuals were able to walk for 20 minutes without a break; otherwise, the 20% who did not have the same performance have a higher level of injury and required rest intervals due to fatigue and pressure on heels. The parameters gait speed, energy cost, stride length, PCF, hip ROM and hip extension VEL showed a strong relevance to the injury level.
Lünenburger et al., 2006 [39].	Pilot study.	CG:<br>● 9 HS.<br>● Mean aged: 29 years.<br>EG:<br>● 13 subjects with SCI.<br>● 3 female and 10 male.<br>● Aged between 21 and 67 years.<br>● Injury level: C4-L1.<br>● AIS A and B.<br>● Time lesion: 0.2 and 33 years.	● Lokomat.<br>● 70% BWS.	It was recorded the EMG activity in differents speeds (0.42 m/s, 0.56 m/s and 0.69 m/s) of the following muscles: BFM, TA, RF, MG during 20 gait cycles, each.	SCI participants presented lower amplitude of EMG signal in BFM, MG and TA and greater amplitude in RF. In addition, the amplitude of EMG signal increase of speed. Further, it was not observed significant differences the comparisons between the electrophysiological signs and HS.
Banz et al., 2008 [40].	Pilot study.	● 12 subjects with incomplete SCI.<br>● 5 female and 7 male.<br>● Injury level: T5-T12.<br>● AIS C and D.<br>● Time lesion: 15 months.	● Lokomat connected to BWS.<br>● Speed was kept constant at 0.55 m/s.<br>● 2 times per week.<br>● 30-40 minutes.	It was observed through graphs analysis the efferent motor level during gait training and the related feedback, primarily, hip and knee extension in the stance phase and hip flexion at the beginning of the swing phase.	During the RAGT it was noticed an increase of motor efference as the participants increased voluntary contribution to the movement. It was observed changes in the hip joint during the stance and swing phases, whereas minimal changes were observed for the knee. Furthermore, 11 participants reported that visual feedback reflected in a better performance during the training.

Moreh et al., 2009 [41].	Case study.	● 1 subjects with syndrome of Brown-Sequard.<br>● Injury level: T11 to right.<br>● AIS C.	● It was performed conventional physiotherapy and RAGT two times per in a total 18 sessions.<br>● Lokomat.<br>● BWS at 60% at the beginning progressing to 0% at the end.	● Magnetic resonance imaging evaluation.<br>● Ashworth scale.<br>● SCIM.<br>● Berg scale.<br>● WISCI II.	At the beginning of the study the participant was AIS C and at the end progressed with AIS D. Further, the rehabilitation period was concluded with muscle strength in the lower limbs improvement to 3-4/5 on the right leg and 4-5/5 on the left leg. Thus, the participant reached a total level of independence for transfers and basic daily lives activities, presenting a score of 90 in SCIM. In addition, it was able to ambulate independently with crutch assistance for long distances and climb stairs. The score obtained during the evaluation of Berg scale was 56, while in WISCI II the scored increased to 15, suggesting that RAGT is effective for treatment of subjects with Brown-Sequard syndrome, and its effect can be enhanced associated to conventional physiotherapy. This may be justified by neuroplasticity in the spinal cord central pattern generators.
Manella, Torres and Fotes, 2010 [42].	Case study.	● 1 subject with SCI.<br>● Male.<br>● Injury level: T7.<br>● AIS A.<br>● Time lesion: 2 years.	● LEMS.<br>● Pendulum test of quadriceps spasticity.<br>● AIS sensory scores.<br>● Functional independence measure (FIM).	● Lokomat.<br>● BWS of 80%.<br>● 3 times per week in a total of 33 sessions (12 weeks).<br>● 40 minutes.<br>● Treadmill speed was increased from 2.22 to 8.89 m/s.	Initially, the participant presented a sensibility absent below T8 and at the end of the intervention below T12. In addition, prior the study it was scored AIS A and at the end, it progressed to AIS C. The abdominal, paravertebral, psoas and hip flexors muscles strength was evaluated by LEMS and ranged from 0/50 to 4/50, and reduced quadriceps spasticity in lower limbs. Initially, the participant was unable to maintain its posture in sitting, standing positions, whereas at the end of the protocol procedure, with the aid of parallel bars, the patient became able to maintain 60 seconds in seated, and 20 seconds in the standing position. At pre-treatment evaluation, the participant was unable to perform gait. However, at the end, the participant was able to walk for a distance of 3 meters with parallel bars assistance.

Houldin, Luttin and Lam, 2011[29].	Clinical trial.	CG:<br>● 17 HS.<br>● Mean aged between 22 and 75 years.<br>EG:<br>● 9 subjects with SCI.<br>● 2 female and 7 male.<br>● Aged between 22 and 66 years.<br>● Injury level: C4-T11.<br>● AIS D.<br>● Time lesion: 0.8 and 13 years.	● Lokomat.<br>● BWS of 10, 40 and 60%.<br>● 20 steps at a constant speed of 0.5 m/s.	● It was recorded EMG activities at RF, BFM, TA and MG.<br>● It was used to record hip and knee joint angles the Lokomat position sensors and a twin-axis electrogoniometer. The ankle angle was only obtained in four SCI and five control participants.<br>● For detection of foot contact and toe-off times it was used, the FSR placed under the foot heel and hallux.<br>● Kinematic data was measured by the calculation of peak flexion angle of the hip and knee, peak of ankle dorsiflexion angle, and peak of foot trajectory height in the swing phase of each step.<br>● At the end, it was used the 10MWT and the WISC II as parameters of overground locomotor capacity in SCI participants.	It was observed an increase of EMG activity in RF during the swing phase when compared basal values with the values after resistance, peak hip flexion and angle during swing decreased during steps taken against resistance. Although no overall increase in RF EMG activity was observed when adding the resistance, it was observed in CG an alteration in RF muscle activity in the loading application. When the load was removed, it was observed that the CG increased in foot trajectory height associated with hip and knee flexion in the balance phase, whereas in the SCI group an increase in the length of the gait associated with increased hip flexion.
Mirbagheri, Patel and Quiney, 2011 [6].	Pilot study.	● 12 incomplete SCI subjects.<br>● It was examined with different degrees of spasticity.	● Lokomat.<br>● 3 times per week.<br>● 45 minutes.<br>● 12 weeks.	● It was performed the trial 3 times and recorded the average of these trials.<br>● Potentiometer measured joint position, the torque transducer measured torque and the tachometer measured velocity.<br>● Bipolar surface electrodes allowed the record of TA and GS.<br>● Muscle strength was recorded by the MVCs during ankle DF and PF.	It was observed a reduction of intrinsic stiffness from 15% to 45% after 2 and 4 weeks of Lokomat training, as well as 40% reduction of reflex stiffness after 4 weeks Lokomat training. The ankle MVCs for flexor and extensors significantly increased up to 180% and 93%, respectively. Thus, this rehabilitation program suggests a potential to modify the neural circuits responses capable of altering the neuromuscular properties.

Schwartz et al., 2011 [7].	Quasi experiment; single experimental group with matched historical control.	● 28 subjects with SCI.<br>● 227% female and 63% male.<br>● Injury level: cervical, thoracic and lumbar.<br>● AIS A, B, C and D.<br>● Time lesion: 13 and 367 days.	● SCIM.<br>● WISCI II.<br>● FAC.	Two groups received conventional physiotherapy.<br>● 30-45 minutes.<br>● 5 times per week.<br>● Bobath principles.<br>EG:<br>● Lokomat.<br>● 2 or 3 times per week.<br>● 30 minutes.<br>● Speed between 0 and 0.83 m/s.<br>● At the beginning of the treatment, it was applied a BWS of 50%, which was decreased approximately 10% by the session in accordance with participant tolerance without knee collapse or toe drag.	Participants presented a significant improvement in gait ability without statistically significant difference between the groups, according to FAC and WISCI II. In contrast, the study group participants presented better results. In the evaluation of the functional and neurological recovery, statistically significant changes were observed when compared to the pre and post-treatment evaluation. In addition, a significant change was observed when comparing the RAGT group and the control group for the motor and locomotors domains of SCIM.
Knikou, 2013 [36].	Pilot study.	● 14 subjects with SCI.<br>● 4 female and 10 male.<br>● Mean aged: 39 years.<br>● Injury level: C5-T10.<br>● AIS A, B, C and D.<br>● Time lesion between 0.5 and 8 years.	● Lokomat.<br>● 45 sessions.<br>● 5 days per week.<br>● AIS A-B participants, first session:<br>● BWS: 60%.<br>● Speed: 0.44 m/s.<br>● AIS C-D participants, first session:<br>● BWS: 40%.<br>● Speed: 0.55 m/s.<br>● In the subsequent sessions, the BWS was diminished by 5% and the speed increased by 0.02 m/s.<br>● If quadriceps and triceps sural strength increased by a full score, the BWS was diminished 10% in each session. The ankle braces straps were loosened or tighten based on the TA muscle strength, which was assessed every 3-5 training sessions.	● Pre and posttest measurements: treadmill speed, BWS, GF, frequency spasms, step duration, 6MWT, WISCI, TUGT, and finally were grouped based on the AIS, the total of sit-to-stand repetition within 30 seconds.<br>● The participants that presented AIS D performed TUGT and 6MWT.	The increase of walking speed coincided with the decrease of BWS, suggesting improvements of the participants' ability to step (diminished step duration of the right leg and frequency of spasms) due to decreased leg GF by the Lokomat. It was not observed significant differences in the measurement of 6MWT related walking overground ability after training. An identical result was found in the TUGT scores in AIS D participants.

Fenuta and Hicks, 2014 [50].	Pilot study.	● 7 subjects with SCI.<br>● Mean aged: 42 years.<br>● AIS C and D.<br>● Time lesion of 4 years.	● All the subjects used the same amount of BWS determined at the beginning session in the ZeroG.<br>● Subjects supported 3 walking sessions in BWS (between 30% and 41%).<br>● Andago GmbH treadmill system (with or without Lokomat) or overground ZeroG.<br>● Subjects concluded walking trials in a randomized order.	● EMG evaluation associated with L- Force: muscles BFM, RF, MG and TA.	The feedback system of the device may give an advantage to the therapists as the increase of BFM activation on the treadmill-based exercise and the ability to conduct longer sessions using robotic device due to the decrease of cardiovascular and muscular demands imposed on the subject.
Aach et al., 2014 [12].	Pilot study.	● 8 subjects with SCI.<br>● 2 female and 6 male.<br>● Aged between 36 e 63 years.<br>● Injury level: T8-L2.<br>● AIS A, B and C.<br>● Mean time lesion: 19 years.	● HAL.<br>● BWS of 50%.<br>● 5 times per week.<br>● 90 days.	● EMG activity: BF, RF, TA and GM.<br>● Perimetry to assess knee volume.<br>● Ashworth scale.<br>● WISCI II.<br>● LEMS.	The speed on the treadmill changed from 0.91 m/s to 1.59 m/s. Initially, treadmill training time was on average 12.37 min and at the end of 31.97 min and the travelled distance from 195.9 m to 954.13 m. Although the increase in WISCI II was not significant, 3 individuals showed improvement in gait skills. The LEMS increased significantly from 21.75 to 24.38. The muscle volume increased between 5 mm and 50 mm. Finally, 1 patient presented in Ashworth 4 prior workout and at the end 2.

Chishmol et al., 2014 [13].	Pilot study.	● 2 subjects with SCI.<br>● Aged between 30 e 31 years.<br>● Injury level: C5 E T5-T6.<br>● AIS C.<br>● Time lesion between 9 and 12 years.	RAGT:<br>● Lokomat.<br>● 12 weeks.<br>● First stage:<br>● 3 times per week.<br>● Home sensory stimulation on the surface of the tongue of moderate to high intensity twice a week.<br>● Second stage:<br>● The sensorial stimulation changed to 5 times per week.<br>● RAGT in the Lokomat was performed in 6 sections of 5 minutes with a rest between the sections.	Assessment was performed in 3 stages: pre-training (T0), after the first 12-weeks of the lab training (T1), and follow-up (T2) after 12-weeks of training at home.<br>● ABC scale.<br>● 10MWT.<br>● 6MWT.<br>● SCI-FAP.<br>● LSQ.<br>● IPAQ.<br>● FIM.<br>● SCIM.	The task-specific rehabilitation strategy and sensory stimulation showed beneficial changes to potentiate neuroplasticity, improve balance, distance and walking speed, recovery of locomotor function, functional ambulation and quality of life.
Labruyère e Hedel, 2014 [43].	Clinical trial.	● 9 subjects with incomplete SCI divided into two groups:<br>● G1: 5 subjects.<br>● G2:4 subjects.<br>● 2 female and 6 male.<br>● Aged between 41 e 69 years.<br>● Injury level: C4-T11.<br>● AIS C and D.<br>● Mean time lesion: 13 and 189 years.	● G1: RAGT during 4 weeks (16 sessions), followed by more 4 weeks (16 sessions) of muscle strengthening training.<br>● G2 receive the treatment in inverse order of G1.<br>● RAGT: Lokomat and BWS approximately 30% during 45 minutes at speed 1-2 km/h and 2 intervals of 1-2 minutes. The muscle strengthening training included 10 minutes of bicycle warm-up, 4-6 lower limbs strength exercises with 3 sets of 10-12 repetitions and 70% of MVC.	● 10MWT.<br>● WISCI II.<br>● LEMS.<br>● PCI.<br>● Mini- Mental.<br>● VAS.<br>● Berg balance scale.<br>● SCIM.<br>● UEMS.<br>● FET.<br>● FES-1.	It was not observed significant variation in FET, LEMS and SCIM at the beginning and at the end of the interventions and between the groups. The reduction of pain was greater after muscle strengthens than RAGT. The RAGT: the participants travelled 1731 m per session resulting in an increase of resistance in all strengthening exercises. In addition, it was observed progression in the maximum speed reached by the individuals of both groups during RAGT, but no significant differences between the interventions were observed.

Sylos-Labini et al., 2014 [18].	Clinical trial.	CG:<br>● 6 HS.<br>● Aged between 21 and 36 years.<br>EG:<br>● 4 subjects with SCI.<br>● 5 female and 1 male.<br>● Aged between 19 and 43 years.<br>● Injury level: T7-L1.<br>● AIS A and D.<br>● Time lesion between 5 and 49 months.	● Mindwalker.<br>● CG was recorded 4 experimental conditions in the same session:<br>● EXO-assisted.<br>● EXO-unassisted.<br>● NW slow (normal slow walking without exoskeleton).<br>● NW self-selected (normal walking at self-selected speed without the exoskeleton).	EMG activity on the right side: VM, BF, RF, ST, TA, SOL, MG, DELTa, DELTp, FCU and ECU.	The CG performed the balance phase of the EXO-assisted and EXO-unassisted conditions in the same duration. In contrast, the gait speed in these conditions were distinct with higher speed for EXO-unassisted during dead time in stance, whereas in EXO-assisted walking, it was required to move the trunk and trigger the swing step by step. Further, it was observed similar amplitude of the hip and knee joint angular movements in the sagittal plane. However, to trigger the swing phase during assisted walking it was required lateral trunk movements resulting in larger hip abduction. In the EMG activity, during the gait ,EXO-unassisted presented higher activity in comparison to EXO-assisted, primarily in the muscles VM, TA, RF, MG and SOL. At the beginning of stance phase during not-assisted walking, it was not observed activity in BFM and ST with different EMG waveforms. Similarities are observed in the correlation analysis with EMG waveforms for the muscles VM, MG, RF and SOL and differences for the muscles ST, TA and BFM between these two conditions. The SCI participants showed lower gait speed with angular movements during the balance phase. The peak-to-peak amplitude of the exoskeleton torque presented similarities, although the knee torque was larger, suggesting that the main forces for stepping in both groups were provided by the exoskeleton. Moreover, SCI participants used more upper limb muscles (DELTp and ECU) for stepping, although presented a variability of upper limb muscles recruitment when compared to CG. EMG activity in the lower limb muscles was typically minor in any SCI patients, though one SCI patient demonstrated consistent activity in the BF, ST, RF, and MG muscles during the swing phase and beginning of stance.
Niu et al., 2014 [44].	Single-center, unblinded, and randomized study.	CG:<br>● 6 paraplegics and 14 tetraplegics.<br>● Mean aged: 49.7 years.<br>● Time lesion of 7.5 years.<br>EG:<br>● 40 subjects with SCI.<br>● 13 female and 27 male.<br>● Mean age: 42.2 years.<br>● Time lesion of 8.9 years.	● Lokomat.<br>● 4 days per week.<br>● 45 minutes.<br>● Speed of 0.94 m/s.	● Lokomat.<br>● 4 days per week.<br>● 45 minutes.<br>● Speed of 0.94 m/s.	The muscle weakness of TA and gastrocnemius may be related to gait deficit due to its role associated with neuromuscular properties during ambulation. The spasticity of the plantar flexors has an inhibitory effect on the TA activity, and, in addition, causes the spastic hyperactivity of gastrocnemius and hypoactivity of TA to interfere in the capacity of voluntary contraction of these muscles. In addition, beyond improving walking ability, Lokomat training can significantly reduce the neuromuscular changes associated with spasticity and may result in an increase in MVC of the ankle muscles.

Hartigan et al., 2015 [28].	Clinical trial.	● 16 subjects with SCI.<br>● Aged between 13 and 38 years.<br>● Injury level: C5-L1.<br>● AIS A, B and C.<br>Time lesion between 18 and 51 years.	The gait training sessions were included in days 2, 3 and 4, which participants learned to stand and walk using the Indego with appropriate support proposed by the physical therapist.	The gait training sessions were included in days 2, 3 and 4, which participants learned to stand and walk using the Indego with appropriate support proposed by the physical therapist.	It was observed an increase since the alterations were not restricted to the cortico-spinal tract, as it also extends the sensory and S1 cortex pathways. There were also significant differences between the results of initial and post-treatment functional tests (TUGT, 6MWT and 10MWT and LEMS) of the SCI and HS group. Despite functional and excitability changes in S1, there was no significant correlation between these parameters.
Lam et al., 2015 [30].	Double-blind, stratified, randomized controlled trial design.	GC:<br>● 7 subjects with SCI.<br>● Aged between 26 and 55 years.<br>● Injury level: C2-T7.<br>● AIS C and D.<br>● Time lesion between 2 and 20 months.<br>EG:<br>● 8 subjects with SCI.<br>● Aged between 28 and 60 years.<br>● Injury level: C1-T10.<br>● AIS C and D.<br>● Time lesion between 2 and 20 months.	● 45 minutes.<br>● 3 times per week.<br>● 3 months.<br>● CG: Lokomat training without resistance.<br>● EG: Lokomat training with resistance.<br>● BWS established was the minimum tolerated by each participant with initial speed of 1 km/h and gradual increase 0.1 km/h according to the participant's capability of resist in the treadmill.	● 45 minutes.<br>● 3 times per week.<br>● 3 months.<br>● CG: Lokomat training without resistance.<br>● EG: Lokomat training with resistance.<br>● BWS established was the minimum tolerated by each participant with initial speed of 1 km/h and gradual increase 0.1 km/h according to the participant's capability of resist in the treadmill.	During the training, all subjects reported some symptom related to autonomic dysreflexia. The BWS decrease was significant in both groups when comparing at the beginning and at the end of training, although was not significant between the groups. In addition, it was shown a small increase in treadmill speed in both groups. In the Loko-R group the treadmill, speed was significantly lower ($p = 0.001$) and significantly increased from the first to the last week of training ($p < 0.001$) and presented a significant improvement in the SCI-FAP at post-training when compared to the CG.
Sczesny- Kaiser et al., 2015 [25].	Clinical trial.	GC:<br>● 11 HS.<br>● Mean aged: 27.8 years.<br>EG:<br>● 11 subjects with SCI.<br>● 4 female and 7 male.<br>● Mean aged: 46.9 years.<br>● Injury level: T7-L3.<br>● AIS A, B and C.<br>● Time lesion of 8.8 years.	● HAL.<br>● 30 minutes.<br>● 5 times per week.<br>● 12 weeks.	● It was recorded the EMG activity of hip and knee extensors and flexors.<br>● Comparison of excitability levels of HS, it was evaluated ppSEP.	It was observed an increase in excitability and cortical representation since the alterations were not restricted to the cortico-spinal tract due to extends the sensory and S1 cortex pathways. Despite functional and excitability changes in S1, there was not a significant correlation between these parameters.

Donati et al., 2011 [11].	Pilot study	● 8 subjects with SCI.<br>● 2 female and 6 male.<br>● Aged between: 26 and 38 years.<br>● Injury level: T4-T11.<br>● AIS A and B.<br>● Time lesion between of 3 and 13 years.	At the end, the total of sessions was 2,052 equivalent to 1,958 hours. The protocol consisted of conventional physiotherapy and BMI application paradigms contemplated 6 items:<br>● Patient seated inserted in a virtual environment, using its own brain activity (captured by 16 EEG channels).<br>● Immersion in virtual environment and similar BMI protocol while the patient was standing by a stand-in- table device.<br>● Training on Lokomat.<br>● Training with a BWS gait system fixed on an overground track by ZeroG.<br>● Training with a brain-controlled robotic BWS gait system on a treadmill.<br>● Gait training with a brain-controlled, sensorized 12 DOFs robotic exoskeleton.<br>● Only in items 3 and 4 individuals did not receive tactile-visual feedback.	On the first day of patients, training started the clinical assessments (Day 0), which was repeated after, 4, 7, 10 and 12 months.<br>● EEG and EMG analysis.<br>● AIS scale.<br>● Semmes-Weinstein Monofilament Test, temperature evaluation, vibration, deep pressure sensitivity, proprioception.<br>● L-force and L-stiff Lokomat.<br>● VAS.<br>● McGill Pain Questionnaire.<br>● ROM of lower limb joints (Medical Research Council Scale).<br>● Modified Ashworth Scale.<br>● BDI.<br>● EMG analysis included the following muscles RF proximal portion, RF distal portion, GM, long toe extensors, gluteus medius, gluteus maximus, hip adductor, medial and lateral hamstrings, and long toe flexor (proximal and distal).	● EEG assessment, after 8 to 10 months a significant EEG desynchronization was observed.<br>● The evolution of motor function was also quantified through multi channel surfing EMG recordings after the 7th and 12th month of treatment. The patients began to present EMG activity below the level of the lesion in at least one muscle, after 7 months of treatment, and after the 12th month, this evolution presented more significant.<br>● The muscles that exhibited the best recovery were: the gluteus maximum, gluteus medius, RF, hip adductors, MH and LH, sural triceps and TA.<br>● Further, according to AIS, showed an improvement in voluntary muscle contraction below the injury level, especially in the proximal muscles.<br>● It was observed improvements in seated and static and dynamic balance.<br>● The somatosensory evaluation throughout this protocol did not show a significant improvement in temperature sensitivity, but the sensitivity to pressure started to show changes between the 4th and 10th month. The vibration sensitivity also showed positive evolutions in the same period in the hip joints (anterior superior Iliac spine), knee and ankle, proprioception improved significantly between the 4th and 12th month, mainly at the level of hip flexion and extension of both limbs and between the 7th and 12th month at knee and ankle level.<br>● It was observed an improvement in all participants of the maximum voluntary contraction evaluated by L-Force in Lokomat, ranging from 0.8 to 21.14 Nm. In addition, it was noteworthy that the motor recovery occurred from proximal to distal, with greater evidence in the hip joint.<br>● According to the rehabilitation progression, the score attributed to the self-report of pain intensity decreased by an average of 2-3 as measured by the VAS, and the perception of pain at the moment of the evaluation decreased during all year.<br>● During the treatment protocol, all participants reported some type of pain sensation below the level of the lesion, although it was difficult to report the exact location of the pain, as measured by the McGill questionnaire.

- In concordance of this sensorimotor recovery, in 12 months of treatment 3 patients changed the classification of AIS from A to C and 1 patient changed from AIS from B to C.
- At the beginning of treatment, all patients preserved ROM and grade 2 spasticity, according to the Ashworth scale. At the end of 12 months, they maintained preserved ROM without muscle contractions and reduced spasticity, according to the L-stiff test - Lokomat.
- In concordance of this sensorimotor recovery, in 12 months of treatment 3 patients changed the classification of AIS from A to C and 1 patient changed from AIS from B to C.
